# Exercise-Induced Irisin: A Novel Strategy for Neuroinflammation Alleviation and Neurorepair in Diabetic Retinopathy

**DOI:** 10.3390/ijms27041849

**Published:** 2026-02-14

**Authors:** Hanlai Song, Yuxian Jiang, Shun Zhang, Chenmian Wu, Chaohua Deng, Weikun Hu

**Affiliations:** 1 Department of Ophthalmology, The First Clinical College, School of Medicine, Wuhan University of Science and Technology, Wuhan 430064, China; hanlaisong1867@163.com (H.S.); 13720119162@163.com (S.Z.); 2 Department of Ophthalmology, Tongji Hospital, Tongji Medical College, Huazhong University of Science and Technology, Wuhan 430030, China; jiangyuxian1204@163.com (Y.J.); wcmleon@gmail.com (C.W.)

**Keywords:** physical exercise, irisin, diabetic retinopathy, ferroptosis, inflammation, neuroprotection

## Abstract

Diabetic retinopathy (DR) stands as a classic microvascular complication of diabetes mellitus. DR is characterized by multidimensional pathological changes in retinal neurons, microvasculature and supportive cells, leading to an intricate damage network. It is predominantly marked by neuropathy, encompassing retinal neuronal dysfunction, aberrant activation of glial cells, and degeneration of synaptic structures. In severe instances, it can result in visual impairment and, in the worst-case scenario, blindness. As diabetes progresses, retinal nerve tissue frequently sustains damage owing to oxidative stress, inflammatory responses, and compromised mitochondrial function. Although the precise neuroprotective mechanisms remain elusive, exercise has the ability to bolster mitochondrial function in retinal cells, diminish oxidative stress, and curb inflammatory reactions, thereby safeguarding the neurophysiological function of the retina. Irisin is a myokine primarily secreted by skeletal muscles in response to exercise stimulation. Moreover, being produced in trace amounts across a variety of tissues, it has the capacity to regulate the physiological processes of multiple organs. Recent studies have indicated that irisin can exert powerful neuroprotective effects by enhancing cellular glucose uptake, improving mitochondrial function, inhibiting the expression of pro-inflammatory factors, and resisting ferroptosis. In this review, we systematically collated and synthesized existing evidence on irisin-related signaling pathways and comprehensively assessed its regulatory potential in alleviating neuroinflammation and promoting neural repair in diabetic retinopathy and offer insights into future research directions in this field.

## 1. Introduction

Diabetic retinopathy (DR), the most common microvascular complication of diabetes, is characterized by retinal microvascular damage and neovascularization. It represents a leading cause of visual impairment in individuals with diabetes. According to World Health Organization (WHO) research, the number of people with diabetes is projected to rise to 578 million by 2030 and 700 million by 2045. Globally, the prevalence of DR among diabetic patients is 22.27% [1,2,3].

DR exhibits multidimensional pathological changes, and understanding this complexity requires recognizing the retina’s highly ordered, sophisticated architecture. The neuroretina, a transparent neural tissue in the innermost layer of the eyeball, adheres closely to the retinal pigment epithelium (RPE); it comprises nine layers from the vitreal to choroidal side, together forming the widely cited ten retinal layers with the RPE. The internal limiting membrane (ILM)—a basement membrane formed by fused Müller glial cell end-feet—acts as a physical barrier, provides structural support, and separates the neural retina from the vitreous body. The nerve fiber layer (NFL) consists of unmyelinated ganglion cell axons that converge at the optic disc to form the optic nerve, and its extreme thinness in the macula minimizes obstruction to photoreception. The ganglion cell layer (GCL) contains ganglion cell somata, the retina’s output neurons, while the inner plexiform layer (IPL), a pure synaptic layer, mediates signal integration. The inner nuclear layer (INL) houses somata of various interneurons and Müller cell nuclei, serving as the core for initial visual signal processing. The outer plexiform layer (OPL), another synaptic layer, facilitates signal transmission between photoreceptors and interneurons and contains most capillaries of the outer retina. The outer nuclear layer (ONL) is a dense cluster of cone and rod nuclei, sustaining the metabolic homeostasis of photoreceptors, whereas the external limiting membrane (ELM)—composed of adherens junctions—provides structural support and separates nuclear layers from photoreceptor layers. The cone-rod (photoreceptor) layer has outer segments rich in visual pigments: cones, concentrated in the macular fovea, govern photopic and color vision, while rods, dominant in the periphery, mediate scotopic vision and dim light perception. As a non-neural component, the RPE is a melanin-containing cuboidal epithelial monolayer that phagocytoses shed photoreceptor membranous discs, transports nutrients and waste, absorbs scattered light, and constitutes the critical outer blood–retinal barrier (BRB) [4].

Under diabetic conditions, the neural retina is highly vulnerable to chronic hyperglycemia. Metabolic dysregulation induced by diabetes, including oxidative stress, the accumulation of advanced glycation end products (AGEs), and persistent inflammation, directly impairs retinal cellular components and disrupts their dynamic homeostasis. Critically, early neuroretinal alterations precede and drive classical microvascular pathology [5]. According to recent research, degenerative alterations such as retinal neuron death, glial cell activation, and neuroinflammatory reactions have already occurred in DR patients before visible vascular abnormalities manifest [6]. These early neural alterations not only constitute a critical initial stage in the pathological progression of DR but also directly influence the prognosis of a patient’s visual function. This challenges the conventional view that focuses solely on vascular pathology. Specifically, retinal ganglion cells (RGCs) and amacrine cells in the inner retina undergo apoptosis, resulting in thinning of the nerve fiber and ganglion cell layers [7,8,9]; photoreceptors are subjected to oxidative stress and subsequent apoptotic loss, further compromising visual function; synaptic connections within the IPL and OPL are disrupted [10,11], impairing visual signal transmission. Additionally, Müller cells undergo reactive gliosis, upregulating the expression of inflammatory cytokines and vascular endothelial growth factor (VEGF), thereby exacerbating neuronal stress and vascular leakage. Microglia become aberrantly activated, shifting toward a pro-inflammatory phenotype that amplifies neuroinflammation and promotes pathological neovascularization. These processes lead to functional deficits such as reduced contrast sensitivity and abnormal electroretinogram responses [12].

Although clinical management of DR has become relatively standardized and includes treatments such as intravitreal glucocorticoid injections, pars plana vitrectomy, retinal laser photocoagulation, and anti-VEGF therapy, these approaches remain predominantly focused on mid- to late-stage vascular complications. By contrast, early neuroprotective interventions still lack demonstrated efficacy, and no effective strategies currently exist to counteract neuroinflammation and neuronal loss in the initial phases of the disease. Consequently, by the time DR is diagnosed, most patients have already progressed to a stage where retinal damage is largely irreversible [13,14,15].

Lifestyle interventions, particularly regular exercise, have demonstrated a clear protective effect against diabetes and its complications. Irisin, a key myokine released in response to exercise, is considered a central mediator of this protection. As type 2 diabetes mellitus (T2DM) represents the primary population affected by DR [16], modifiable risk factors such as obesity are of major clinical relevance. Regular exercise improves glycemic control, blood pressure regulation, weight management, and overall complication risk [17]. Both resistance training and aerobic exercise have proven beneficial in T2DM management [18,19]. Irisin is secreted by skeletal muscle during physical activity. Initially identified by Boström et al. [20] as a factor that promotes browning of white adipose tissue and modulates energy metabolism, irisin has since been recognized for its antidiabetic properties. Subsequent studies indicate that it enhances glucose homeostasis and reduces insulin resistance [21]. Notably, serum irisin levels are decreased in T2DM patients and inversely correlated with the incidence of new-onset T2DM [22,23,24,25]. Beyond its metabolic role, growing evidence suggests that irisin exerts multiple therapeutic effects, including improved cognitive function [22,26,27,28], antifibrotic activity [29,30], inhibition of pathological neovascularization [31], neuroprotection [28,32,33], and prevention of ferroptosis [34]. Therefore, we hypothesize that irisin, by mediating anti-inflammatory, antioxidant, and anti-ferroptotic mechanisms, may serve as a novel therapeutic agent for early intervention in DR.

## 2. Biological Properties of Irisin

### 2.1. Source, Molecular Characteristics and Receptors of Irisin

Irisin was first identified in 2012 as a myokine produced by the proteolytic cleavage of fibronectin type III domain-containing protein 5 (*FNDC5*) [20,35]. Its production is initiated when physical exercise activates skeletal muscle, leading to the upregulation of peroxisome proliferator-activated receptor gamma coactivator 1-alpha (PGC-1)—a master transcriptional coactivator that drives the expression of *FNDC5*. Following translation, *FNDC5* undergoes post-translational modifications and cleavage of its C-terminal transmembrane segment, releasing a soluble 112–amino acid N-terminal fragment known as irisin.

Structurally, irisin is a protein hormone with a molecular mass of approximately 12,000–12,587 Da. Its amino acid sequence is highly conserved among mammals; human and mouse irisin, for example, share 95.83% identity, underscoring its evolutionary preservation of essential biological roles. The active peptide is derived from the N-terminal fibronectin type III (FNIII) domain of *FNDC5* and can form a dimer in vivo. This dimerization enhances receptor binding by optimizing the ligand–receptor interface, thereby amplifying downstream signaling and biological activity [36].

The biological activity of irisin and its precursor *FNDC5* is precisely regulated by post-translational modifications, which collectively determine the generation, intensity, and functional specificity of irisin signaling. Phosphorylation acts as the core mechanism underlying irisin signal transduction; as a primary upstream activator, irisin induces the phosphorylation of key downstream kinases such as p38 MAPK [37], JNK [37], AMPK [38], ERK [39], and STAT4 [40], thereby activating multiple signaling pathways and playing a central role in metabolic regulation [39], cell differentiation [37], and cytoprotection, and it can also modulate the phosphorylation status of target proteins (such as the glucocorticoid receptor) to exert direct biological effects [41]. Glycosylation mainly regulates the bioavailability of irisin and *FNDC5*: N-glycosylation of the *FNDC5* protein at Asn36 and Asn81 is critical for its stability, proper processing, and the secretion of active irisin [42,43], which constitutes a prerequisite for its effective biosynthesis, while to date, there is no evidence that *FNDC5* or irisin itself undergoes O-GlcNAcylation. Notably, O-GlcNAcylation is often hyperactivated under metabolic stress, and its potential association with the irisin system remains an uncharted research area. In summary, phosphorylation precisely controls the “switch” and pathway selection of irisin signaling, while glycosylation ensures its stable “output,” and together, these two post-translational modifications synergistically shape the overall biological activity of irisin.

The tissue distribution of irisin is far more extensive than initially recognized. Although first identified in mouse skeletal muscle [20], subsequent research has confirmed its presence in a wide variety of tissues and biological fluids [44]. In humans, *FNDC5* mRNA is expressed not only in skeletal muscle but also in numerous other organs and structures, including the brain, heart, liver, kidneys, retina, spinal cord, thyroid, adrenal glands, pituitary gland, ovaries, testes, and various components of the digestive, circulatory, and reproductive systems. Irisin or its precursor has also been detected in plasma, cerebrospinal fluid, saliva, and breast milk [45,46,47]. Similarly, in rodents, both *FNDC5* mRNA and irisin protein have been found in adipose tissue, cardiomyocytes, the hippocampus, and other non-muscular sites [48,49]. This broad expression pattern strongly suggests that irisin’s physiological functions extend well beyond the regulation of energy metabolism.

The exertion of irisin’s physiological functions relies on its binding to specific receptors. Extensive existing research has demonstrated that integrin αVβ5 is the key functional receptor mediating irisin’s effects in various tissue cells. This receptor is widely expressed across multiple tissues, including the intestines, nerves, pancreas, heart, adipose tissue, muscles and eyes [33,49,50,51,52,53]. Specifically, in the retina, its expression has been verified in vascular endothelial cells [54], RPE cells [55], retinal microglia [56], and astrocytes [57]. These findings suggest that irisin may help maintain retinal homeostasis through neuroprotective, metabolic, and vasoprotective mechanisms. However, the precise signaling pathways, upstream regulators, and clinical potential of irisin in the retina require further systematic investigation.

### 2.2. Regulation of Irisin Secretion by Exercise

Different modes of exercise differentially regulate irisin secretion, yet they all converge on the core PGC-1/*FNDC5* signaling axis. Exercise activates the AMPK/PGC-1α pathway, which upregulates *FNDC5* expression and enhances its proteolytic cleavage to release irisin. Among various exercise types, endurance exercise, acute exercise, and resistance exercise have different effects on irisin secretion.

Endurance exercise, characterized by sustained rhythmic physical activity, has been shown in numerous studies to regulate irisin secretion, with significant heterogeneity in the intensity of this regulatory effect (Table 1). The core influencing factors include exercise intensity, exercise duration, and individual characteristics of participants such as age [58]. There is a consensus in clinical and basic research that moderate-intensity aerobic exercise is a reliable means of inducing irisin production: animal experiments have demonstrated that 4 weeks of swimming training significantly increases plasma irisin concentrations in both rats and mice [59]; studies in both humans and animals have confirmed that sustained moderate-intensity aerobic exercise can upregulate irisin expression, simultaneously increasing its circulating concentration and local synthesis in skeletal muscle [60,61,62]; population studies have also found that the blood irisin concentration in long-term endurance exercisers is twice that in sedentary individuals [20]. Discrepancies in existing research mainly focus on the regulatory roles of exercise intensity and age, which are also the core variables leading to result heterogeneity. Studies on healthy, untrained middle-aged men have shown that high-intensity endurance training does not significantly alter the expression levels of PGC-1α and *FNDC5* in skeletal muscle or serum irisin concentrations [63]. This negative result is associated with three factors: reduced physiological responsiveness of middle-aged men to high-intensity exercise, methodological differences in training protocols including variations in training frequency and recovery periods, and technical limitations in experimental procedures including differences in detection sensitivity and deviations in blood sampling timing. Additionally, rigorous controlled studies have confirmed that age is a key regulatory factor in irisin response—8 weeks of endurance training significantly increases serum irisin levels in healthy middle-aged and elderly participants, while no statistically significant effect is observed in younger participants [64]. These findings offer a targeted framework for irisin’s clinical and experimental use, advising moderate-intensity aerobic exercise for older adults and individualized high-intensity training parameters for younger populations; standardizing experimental and training protocols is also critical to boost research reproducibility, translational value and support the clinical and experimental advancement of irisin interventions.

Acute exercise elicits variable effects on serum irisin levels (Table 2). Some studies have shown that there is no significant difference in serum irisin levels between the exercise group and the control group after high-intensity interval training (HIIT) and resistance training [65]; however, other studies have reported that acute exercise can significantly increase circulating irisin levels [66,67]. The mode of exercise appears critical: for instance, high-intensity exhaustive running stimulates irisin release during and after exertion, whereas an equivalent cycling protocol does not produce a similar response [68]. Rope skipping—a form of HIIT—has been found to significantly enhance irisin secretion, particularly in obese adolescents. In one intervention, combining rope skipping with dark chocolate supplementation not only elevated serum irisin but also improved body composition and reduced inflammatory markers [69]. In addition, serum irisin levels in football players are positively correlated with whole-body bone mineral density (TB-BMD), and football matches can significantly increase irisin levels at different time periods (morning, afternoon, and evening), and this effect is consistent in all subjects [70,71]. Similarly, a single 20-s all-out bicycle sprint led to significant increases in irisin at 15, 30, and 60 min into recovery [72]. At the molecular level, Continuous HIIT has been shown to upregulate *FNDC5* mRNA expression in skeletal muscle [73]; however, this transcriptional activation does not always translate into detectable changes in plasma irisin levels [74]. Collectively, these findings indicate that not only is acute exercise-induced irisin release largely independent of training status, but it may also be modulated by the specific mode of training.

Resistance exercise can significantly stimulate irisin secretion, although research findings on its effects remain inconsistent (Table 3). Clinically, resistance training may induce a more pronounced irisin response than endurance exercise alone, with combined training yielding the greatest effect [75]. However, the duration and design of resistance interventions appear critical. For instance, an 8-week resistance training program did not significantly alter serum irisin levels in healthy individuals [76], whereas 12 consecutive weeks of resistance training effectively elevated circulating irisin in both mice and humans [77,78]. Acute responses to resistance exercise are similarly variable. One study reported a transient rise in blood irisin one hour after a single high-intensity strength session (e.g., weightlifting), with levels returning to baseline thereafter [79]. In contrast, another study found no significant irisin change despite clear increases in lactate and heart rate following similar resistance exercise [80]. Moreover, Specific forms of resistance training also influence irisin dynamics. High-intensity static exercises targeting the lower limbs and core, such as leg raises, elevate both serum irisin and local *FNDC5* expression in muscle [77]. Similarly, compound bodyweight movements like squats and push-ups can stimulate irisin secretion [81], highlighting the ability of varied resistance modalities to modulate this myokine.

In summary, while irisin levels are modulated by exercise type, intensity, duration, and individual factors such as age and health status, future research should prioritize two key objectives: first, elucidating the mechanisms behind these interindividual differences, and second, translating this understanding into precise, personalized exercise regimens capable of effectively leveraging irisin for the prevention and treatment of metabolism-related diseases.

## 3. Early Neurodegeneration in Diabetic Retinopathy: The Key Therapeutic Target of Irisin

Irisin is an exercise-induced endogenous peptide capable of crossing the BRB. It plays a critical role in modulating neuroinflammation, oxidative stress, ferroptosis, and the homeostasis of the neurovascular unit (NVU) [26,82,83], thereby emerging as a promising therapeutic target for early neurodegeneration in DR.

### 3.1. Neuroinflammation: Abnormal Activation of Microglia

Chronic inflammation is a key contributor to the development of diabetes and its complications [84] and serves as a central driver in the pathogenesis of DR. DR is now recognized as a chronic, low-grade inflammatory state perpetuated by sustained hyperglycemia and metabolic dysregulation. In this pathological setting, it is the high-glucose microenvironment that aberrantly activates retinal microglia (the resident immune cells), leading them to initiate and amplify neuroinflammation.

Hyperglycemia drives microglial activation through interconnected pathways: it induces oxidative and endoplasmic reticulum stress [85], leading to activation of NF-κB and other pro-inflammatory transcription factors; simultaneously, it promotes the accumulation of AGEs, which engage RAGE and further stimulate inflammatory signaling [86,87]. Guided by these signals, microglia shift from a surveillance to an activated phenotype, releasing inflammatory cytokines such as TNF-α and IL-1β, thereby driving a self-perpetuating inflammatory cascade. Notably, irisin has been shown to reprogram microglial activation via the integrin αVβ5 receptor [56]. This interaction suggests a novel therapeutic strategy for mitigating neuroinflammation and BRB dysfunction in DR, whereby irisin may disrupt the pathogenic cascade at the cellular level through the αVβ5/AMPK signaling axis.

In the inflammatory milieu of DR, vascular endothelial cells exposed to a high-glucose environment upregulate adhesion molecules and recruit macrophages, neutrophils, and other inflammatory cells into the retinal tissue. These infiltrating cells coalesce with activated microglia to form localized inflammatory foci. Activated immune cells release pro-inflammatory cytokines, such as TNF-α and IL-1β, which act directly on retinal neurons [88], disrupting their energy metabolism and synaptic transmission, ultimately leading to neuronal dysfunction and apoptosis, which is also an important pathological basis of early neurodegeneration in DR. In parallel, inflammatory factors, together with VEGF and matrix metalloproteinases (MMPs), synergistically disrupt BRB integrity by degrading tight junction proteins, increasing vascular permeability [89,90,91], and promoting retinal edema [92]. Elevated vitreous TNF-α levels in diabetic patients and diabetic rats correlate with DR severity and BRB leakage [93,94]. Moreover, chronic inflammation driven by hyperglycemia induces VEGF overexpression, which stimulates pathological neovascularization [95,96,97] and accelerates the progression of DR from the non-proliferative phase to the proliferative phase.

### 3.2. Oxidative Stress and Ferroptosis

Oxidative stress, a pathological state caused by excessive production of reactive oxygen species (ROS) and impaired antioxidant defenses, plays a central role in the pathogenesis of DR. In the hyperglycemic retinal milieu, mitochondrial dysfunction, particularly in photoreceptors, disrupts the electron transport chain and drives overproduction of superoxide anions, representing a key initiating event in DR [98,99]. Sustained oxidative stress triggers mitochondrial damage, apoptosis, neurodegeneration, lipid peroxidation, microvascular leakage, and inflammatory activation, which jointly promote the progression of DR [100,101]. It is worth noting that oxidative stress does not act in isolation but engages in a self-amplifying loop with major hyperglycemia-induced pathways—including the polyol and hexosamine pathways, AGEs formation, and protein kinase C (PKC) activation—thereby mutually amplifying damage and accelerating the progression of DR [102].

In DR, hyperglycemia activates the polyol pathway of glucose metabolism. Through the utilization of NADPH, glucose is reduced to sorbitol by aldose reductase (AR) in this pathway, a process that increases intracellular osmotic pressure and leads to capillary damage and cell death. Sorbitol is subsequently oxidized by sorbitol dehydrogenase to generate fructose, accompanied by the reduction of NAD^+^ to NADH [103,104,105]. The resulting NADH surplus serves as a substrate for NADH oxidase, promoting further ROS generation in retinal cells and aggravating oxidative stress [106]. Elevated ROS levels inhibit glyceraldehyde-3-phosphate dehydrogenase (GAPDH) activity. Concurrently, increased influx of phosphorylated glucose activates the hexosamine pathway. Glucosamine produced via this pathway enhances hydrogen peroxide (H_2_O_2_) generation, which in turn promotes oxidative stress, endothelial dysfunction, increased vascular permeability, and angiogenesis [107].

High glucose–induced oxidative stress inhibits the activity of GPX4, a key enzyme responsible for neutralizing lipid peroxides, thereby increasing the susceptibility of retinal cells, including Müller cells, retinal pigment epithelial cells, and microglia, to ferroptosis [108,109,110,111]. This mechanism provides a novel explanation for hyperglycemia-mediated retinal injury. Not only does ferroptosis exacerbate oxidative damage in retinal tissue, but it also intensifies inflammatory responses to drive the progression of DR.

### 3.3. Neurovascular Unit (NVU) Injury and Blood–Retinal Barrier Disruption

The retinal NVU is essential for maintaining retinal homeostasis, integrating vascular perfusion with metabolic demand and preserving BRB integrity. Disruption of NVU function and breakdown of the BRB are early and fundamental events in the pathogenesis of DR [112,113], with impairment of the inner BRB (iBRB) serving as a key marker of disease onset [114]. The BRB consists of an iBRB and an outer barrier (oBRB) [115], which together regulate the exchange of substances between the blood and retinal tissue, prevent harmful agents from entering the retina [116], and maintain a stable microenvironment for retinal nerve cells. Chronic hyperglycemia disrupts retinal vascular homeostasis by activating pathways such as the polyol and PKC pathways, thereby establishing the foundation for BRB damage [117] and promoting the progression of DR.

The core of the iBRB is formed by tight junctions (TJs) between retinal capillary endothelial cells [117] and the pericytes that ensheathe them. Early iBRB damage is a hallmark of DR [118]. In a hyperglycemic environment, endothelial TJs are compromised through two major mechanisms. First, activation of the AGEs-RAGE and PKC pathways induces the release of inflammatory factors such as TNF-α, IL-6, and ROS, which degrade TJ proteins or suppress their gene expression [119,120]. Second, high glucose-induced retinal ischemia and hypoxia stimulate Müller cells and astrocytes to secrete VEGF. VEGF binding to endothelial VEGFR2 accelerates TJ degradation and widens intercellular gaps, thereby increasing vascular permeability, promoting extravasation of blood macromolecules, and contributing to vasogenic edema [121,122]. Early pericyte loss compromises iBRB structural and functional integrity. Hyperglycemia induces pericyte apoptosis via the polyol pathway and AGE accumulation, or activates AGEs–RAGE signaling in pericytes to inhibit glucose uptake and trigger cell death [123,124], thereby reducing vascular wall elasticity and increasing permeability. Additionally, chronic hyperglycemia thickens and stiffens the retinal capillary basement membrane [125,126], which impairs nutrient exchange, mechanically stresses endothelial junctions, and indirectly disrupts TJ function, ultimately establishing a self-perpetuating cycle of ischemia and barrier breakdown.

The oBRB consists of the choriocapillaris, Bruch’s membrane, and RPE cells [127,128]. Located between photoreceptor outer segments and the choriocapillaris, it mediates nutrient supply and waste clearance for the outer retina [129,130]. Although oBRB impairment is often subtle in early DR, its dysfunction becomes more pronounced as the disease progresses. RPE cell tight junctions are mainly composed of claudin-19 and ZO-1 [131,132], which is the key to preventing choroidal blood macromolecules from entering the outer layer of the retina. Once the structure of its TJs is destroyed, choroidal substances will abnormally penetrate into the retinal tissue. Hyperglycemia disrupts RPE function [133,134] in several ways. It impairs the phagocytosis of shed photoreceptor outer segments [135], blocking the transport of essential nutrients for photoreceptor cells such as glucose, retinol, vitamin A, and fatty acids, leading to photoreceptor cell degeneration and apoptosis [136,137]. Additionally, hyperglycemia reduces the secretion of neurotrophic factors like pigment epithelium–derived factor (PEDF) [138], diminishing neuroprotection and potentially exacerbating barrier dysfunction through disrupted vascular homeostasis, thereby accelerating DR progression.

### 3.4. Relative Contributions of Retinal Neurons, Microvasculature and Supportive Cells to DR Pathogenesis

DR pathogenesis is best characterized as a dynamic, multicompartmental disorder. The dysfunction of retinal neurons, the microvasculature, and support cells—primarily Müller glia and microglia—evolves temporally in an interwoven manner, creating a synergistic and self-amplifying network of damage. The relative contributions and chronological sequence of these three compartments progressively shift as the disease advances.

In the early stages, neuronal dysfunction serves as the initiating core, with the neural and NVU affected first, typically in a clinically silent manner [139]. Retinal neurons and photoreceptors undergo subtle metabolic stress and oxidative damage before detectable structural changes appear, representing the initial trigger of injury [140]. Concurrently, mild BRB leakage occurs in the microvasculature, and support cells (Müller glia and microglia) become activated, initiating an immune response and collectively fostering a pro-inflammatory microenvironment. At this stage, neuronal injury is the primary driver, whereas microvascular leakage and glial activation represent secondary responses. Functional impairments mainly manifest as reduced contrast sensitivity and abnormal electroretinogram responses [141,142], whereas classical microvascular signs such as microaneurysms and dot hemorrhages emerge later [143]. As DR progresses, a pathological shift occurs. Microvascular pathology becomes the dominant driver, with widespread capillary occlusion and progressive ischemia replacing neuronal injury as the central pathogenic force. This shift propels the disease into the proliferative stage, characterized by pathological neovascularization and ultimately leading to severe vision loss.

This progression involves all three compartments in a coordinated manner. Chronic hyperglycemia acts as the initiating factor, directly inducing metabolic stress and oxidative damage in neurons and photoreceptors while simultaneously injuring microvascular endothelial cells and pericytes, resulting in increased vascular permeability and luminal occlusion. The ensuing ischemia, together with signals released from damaged neurons, potently activates Müller glia and microglia, driving their transition to a pro-inflammatory phenotype. These activated cells release various cytokines, chemokines, and VEGF, which further exacerbate BRB disruption, promote pathological neovascularization [144], and accelerate neuronal apoptosis [145]. Worsening vascular leakage and ischemia, in turn, aggravate neuronal damage and abnormal glial activation, establishing a vicious cycle of “neuronal injury–microvascular occlusion–glial activation” in which the three components mutually reinforce and amplify damage.

In summary, DR is a classic “multicomponent synergistic injury disorder.” Injury to retinal neurons, the microvasculature, and support cells does not occur in isolation but exists within a tightly interconnected, mutually amplifying causal network: neuronal injury is central in the early phase, with the microvasculature and support cells cooperating in damage initiation; in the advanced phase, microvascular ischemia and occlusion become dominant, reciprocally exacerbating neuronal and glial dysfunction. Therefore, pleiotropic intervention strategies capable of simultaneously modulating the function of all three compartments hold greater therapeutic potential. Exercise-induced irisin, with its demonstrated anti-inflammatory, antioxidant, and neuroprotective properties, represents one such multi-target strategy. It promises to act on several key nodes within this pathogenic network, offering simultaneous protection to neurons, stabilization of the microvascular barrier, and regulation of support cell activation.

## 4. Neuroprotective Mechanism of Irisin in Diabetic Retinopathy

### 4.1. Anti-Inflammatory Effect: Regulating Glial Cell Homeostasis

Chronic low-grade inflammation plays a central role in the progression of DR. Hyperglycemia activates multiple inflammatory pathways and promotes the activation of transcription factors such as NF-κB, leading to the release of inflammatory mediators including TNF-α, IL-1β, IL-6, and VEGF. This cascade triggers leukocyte adhesion, vascular leakage, neuroinflammation, and pathological angiogenesis. Irisin exerts potent anti-inflammatory effects and represents a key molecular mechanism underlying the beneficial health effects of exercise. It plays an important role in mediating the protective effects against DR. Specifically, irisin regulates glial cell homeostasis through two primary mechanisms.

Irisin can effectively inhibit core inflammatory pathways such as NF-κB. S Studies confirm that irisin reduces β-cell insulin resistance and inflammation by suppressing the TLR4/NF-κB pathway [146]. This mechanism is also relevant in DR, where irisin downregulates NF-κB activity, thereby reducing the expression of critical pro-inflammatory factors such as TNF-α, IL-1β, IL-6 and VEGF in retinal tissue by down-regulating NF-κ B activity, thereby inhibiting leukocyte adhesion and vascular leakage, modulating the retinal immune microenvironment, and mitigating inflammatory damage to both blood vessels and neurons. Irisin also activates the nuclear factor erythroid 2–related factor 2/heme oxygenase-1 (Nrf2/HO-1) antioxidant pathway, which indirectly enhances its anti-inflammatory actions [147]. Notably, IL-17A has been identified as an independent risk factor for non-proliferative diabetic retinopathy (NPDR) (OR = 1.22). Intriguingly, when irisin was included in the statistical model, the association between IL-17A and NPDR disappeared. Partial correlation analysis further revealed a significant negative correlation between irisin and IL-17A levels (r = −0.252), suggesting that irisin may help prevent DR through a potential anti-IL-17A mechanism [24].

Irisin modulates microglia and macrophage polarization, promoting a shift toward the anti-inflammatory M2 phenotype. Glial cell activation is a major driver of neuroinflammation in DR, and hyperglycemic environment can significantly activate retinal microglia, leading to inflammation [148,149]. Irisin mitigates neuroinflammation by reprogramming activated microglia and macrophages, shifting their polarization from the M1 toward the M2 phenotype [150]. This polarization reduces the release of pro-inflammatory cytokines while enhancing the secretion of anti-inflammatory factors, thereby helping to restore retinal glial homeostasis and attenuate neuroinflammatory damage in DR.

### 4.2. Anti-Ferroptosis and Anti-Oxidative Stress of Irisin

**Figure 1:**
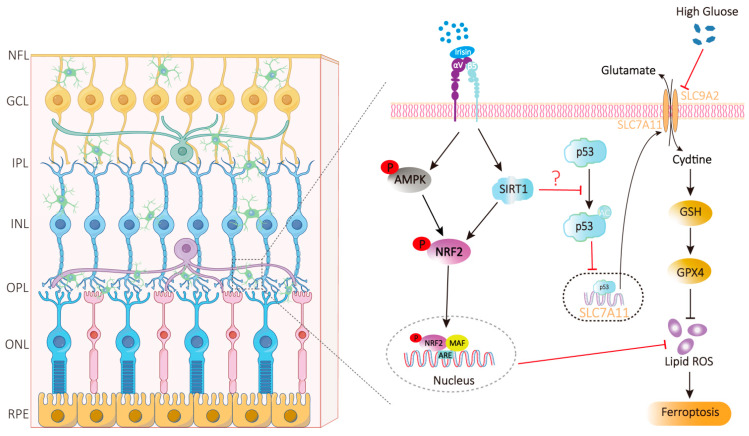
In diabetic retinopathy lies the mechanism through which irisin exerts its inhibitory effect on microglial ferroptosis. (These pathways are largely inferred from research on non-retinal cells; their specific roles in DR have not been conclusively validated.) Under high glucose stimulation, irisin binds to the αV/β3 receptor on the surface of microglia to activate a signal cascade. This cascade regulates glutathione (GSH) synthesis and lipid reactive oxygen species (Lipid ROS) levels through key molecules such as AMPK, NRF2, p53, and SIRT1, ultimately affecting the ferroptosis process. Nerve Fiber Layer (NFL), Ganglion Cell Layer (GCL), Inner Plexiform Layer (IPL), Inner Nuclear Layer (INL), Outer Plexiform Layer (OPL), Outer Nuclear Layer (ONL), and Retinal Pigment Epithelium (RPE). The figure was created with Adobe Illustrator 2026 (v30.1).

Characterized by the pathological accumulation of lipid peroxides, ferroptosis serves as a critical mechanism underlying both cell loss and vascular damage in DR. Hyperglycemia disrupts iron homeostasis in RPE cells and capillary pericytes, leading to excessive ROS generation and lipid peroxidation. In retinal endothelial cells, hyperglycemia significantly elevates levels of free iron, lipid peroxides (LPOs), and ROS, while concurrently suppressing the activity of GPX4 [109]. Irisin mainly exerts anti-ferroptosis and anti-oxidative stress effects through two core pathways (Figure 1).

Irisin inhibits lipid peroxidation by activating the AMPK/Nrf2/GPX4 signaling axis. Irisin can activate AMPK signaling molecules, thereby initiating the activation of nuclear factor E2-related factor 2 (Nrf2) [151]. Once in the nucleus, Nrf2 transcriptionally upregulates antioxidant genes such as GPX4 (a key inhibitor of ferroptosis, which can specifically reduce lipid peroxides), heme oxygenase-1 (HO-1), thereby enhancing cellular antioxidant capacity [152]. By effectively blocking lipid peroxidation, irisin acts through two complementary mechanisms: preserving reduced glutathione (GSH) homeostasis to support GPX4 activity, and modulating iron metabolism to reduce labile iron pools. This protective mechanism closely resembles that of astaxanthin in DR models, where astaxanthin attenuates retinal tissue damage by activating the Nrf2/GPX4 pathway, thereby reducing malondialdehyde (MDA) levels and enhancing GPX4 activity [153]. In models of renal and cardiac ischemia–reperfusion injury, irisin exerts protective effects that are closely associated with a marked reduction in lipid peroxide levels and upregulated expression of GPX4 and superoxide dismutase (SOD) [154,155], further supporting the robustness of this pathway in mediating irisin’s antioxidant actions.

Irisin exerts anti-ferroptotic effects primarily via the SIRT1/Nrf2 or SIRT1-p53-SLC7A11 pathway. Ferroptosis is hallmarked by aberrant expression of SLC7A11 and GPX4, concomitant with elevated MDA levels and reduced GSH content [83,156]. At present, studies have shown that irisin exerts anti-ferroptotic effects by activating the SIRT1/Nrf2 pathway [157]. The upregulation of SLC7A11 expression by irisin is mediated through two key events: the promotion of SIRT1-dependent deacetylation and the downregulation of p53 [83,158]. Through this SIRT1–p53–SLC7A11 axis, irisin upregulates SLC7A11 and GPX4, enhances antioxidant capacity, and reduces MDA levels, counteracting ferroptosis. Although this pathway has not been directly confirmed in the retina, irisin was shown in a type 1 diabetic cardiomyopathy model to reverse ferroptosis-induced increases in MDA and decreases in GSH, SLC7A11, and GPX4 [83]. Given the similar ferroptotic features in DR, this pathway likely contributes to irisin’s retinal protective effects.

### 4.3. Maintain BRB Integrity

Hyperglycemia can destroy BRB integrity through multiple mechanisms such as inflammatory response and ferroptosis. By concurrently targeting both anti-inflammatory and anti-ferroptotic pathways, irisin exerts protective effects on retinal pigment epithelial cells and retinal endothelial cells. This protection contributes to maintaining the tight junctions of the BRB and thereby safeguards the neurovascular units.

Irisin’s protection of the BRB against hyperglycemia-induced damage involves a coordinated network of anti-inflammatory and antioxidant mechanisms. It effectively suppresses the aberrant activation of key pro-inflammatory signaling cascades, specifically the TLR4/MyD88 [159] and NF-κB/MAPK [160] pathways, leading to a marked reduction in the secretion of inflammatory mediators such as TNF-α, IL-6, and IL-17A. In addition, given the structural and functional similarities between the BBB and the BRB, and considering that irisin has been shown to upregulate the expression of tight junction proteins, such as Claudin and Occludin [161], it is plausible that irisin similarly reinforces BRB integrity by modulating these critical junctional proteins. Irisin may also activate the Notch signaling pathway via Notch1 receptor, thereby promoting the repair of high glucose–induced damage in vascular endothelial cells [162].

Irisin may effectively inhibit high glucose-induced ferroptosis in retinal cells, thereby safeguarding both the oBRB and the iBRB. Regarding the oBRB, irisin demonstrates potent anti-ferroptotic and neuroprotective properties in neuronal HT22 cells under high-glucose conditions [163,164]. Given that RPE cells and Müller cells, akin to HT22 cells, originate from the ectoderm and exhibit similar susceptibility to oxidative stress, it is reasonable to hypothesize that irisin could mitigate high glucose or hypoxia-induced ferroptosis in RPE and Müller cells through its well-established anti-ferroptotic mechanisms. This, in turn, would preserve their vital functions, including phagocytosis, trophic support, and ion homeostasis, all of which are crucial for maintaining oBRB stability. Endothelial cells and pericytes of the retinal microvasculature are core structural components of the iBRB and are particularly vulnerable to injury under diabetic conditions. Irisin has been proven to suppress ferroptosis and enhance mitochondrial function in cardiomyocytes and hepatocytes [34,155], indicating its potential to also shield retinal vascular endothelial cells and pericytes from ferroptosis.

Clinical evidence further supports the physiological relevance of these actions. A significant decrease in serum irisin levels, which are negatively correlated with VEGF levels [165], is observed in patients with DR. These findings link low irisin levels to BRB impairment, confirming its key role in preserving barrier function and suggesting its potential involvement in the clinical pathology.

### 4.4. Clinical Correlation Between Irisin Levels and DR Severity

A retrospective study of 3031 adults demonstrated that individuals who maintain high levels of physical activity over the long term have a significantly lower risk of developing DR [166]; Conversely, a sedentary lifestyle markedly increases the likelihood of DR in patients with diabetes [167,168], underscoring the protective role of exercise against DR onset.

Irisin enhances glucose uptake ability in skeletal muscle and adipocytes by activating AMPK and PI3K/Akt signaling pathways, thereby improving insulin sensitivity and reducing blood glucose levels [169,170,171,172]. Studies have confirmed that circulating irisin levels in patients with T2DM are significantly lower than those in healthy controls [173]. Simple regression analysis indicates that in T2DM patients, serum irisin levels are inversely correlated with age, fasting blood glucose, insulin resistance homeostasis model assessment, blood urea nitrogen, and creatinine, and positively correlated with creatinine clearance and ACEI/ARB treatment [23]. Moreover, irisin levels decline further in T2DM patients who develop complications such as diabetic nephropathy or DR [25]. Specifically, in DR-related studies, plasma irisin levels were higher in patients without diabetic retinopathy (No DR) than in those with DR, and higher in patients with NPDR than in those with proliferative diabetic retinopathy (PDR) [174]. Consistently, irisin concentrations in both serum and vitreous humor of PDR patients were significantly lower than those in healthy individuals and in T2DM patients with no DR [23].

### 4.5. The Function of Irisin in Diabetic Complications

Irisin exerts a complex role and holds broad therapeutic promise in T2DM and its associated complications. Clinical studies have confirmed that circulating irisin levels in T2DM patients are lower than those in healthy individuals [23,174,175], while such levels decrease more markedly in patients with concurrent chronic complications [25].

Irisin exerts distinct protective effects against multiple diabetic complications. In diabetic cardiomyopathy, myocardial *FNDC5*/irisin expression and plasma irisin levels are downregulated in T2DM mouse models; exogenous irisin supplementation or *FNDC5* overexpression ameliorates diastolic dysfunction, alleviates myocardial apoptosis, fibrosis, and hypertrophy, largely via activation of the integrin αV/β5-AKT pathway and attenuation of oxidative/nitrosative stress [176]. Serum irisin levels in patients with diabetic nephropathy exhibit a notable decline during the early disease stages [177]. Correspondingly, in db/db diabetic mouse models, genetically mediated upregulation or exogenous administration of irisin can significantly alleviate glomerular injury, renal tubular injury, and renal interstitial fibrosis, ameliorate renal function, and reduce proteinuria [29,177]. A hyperglycemic environment exerts prominent neurotoxic effects on the nervous system; in neuronal cells, hyperglycemia can induce ferroptosis, which is characterized by upregulated ACSL4 expression and elevated levels of iron ions and MDA, while irisin treatment effectively mitigates such neurotoxicity and ferroptosis and enhances cellular antioxidant capacity [163]. Meanwhile, at the cognitive level, hyperglycemia impairs hippocampal synaptic plasticity, whereas irisin confers protection against hyperglycemia-induced cognitive impairment by modulating hippocampal synaptic plasticity via the *FNDC5*/BDNF-Trk axis [178]. In the context of diabetic osteopathy, irisin treatment alleviates osteoblast ferroptosis by inhibiting the endoplasmic reticulum stress-mediated eIF2α/ATF4/CHOP signaling pathway, thereby ameliorating bone loss in type 1 diabetes mellitus (T1DM) mouse models [179]. In contrast, in T2DM mouse models, exercise intervention or exogenous irisin supplementation downregulates oxidative stress-dependent miR-150 (a microRNA that suppresses *FNDC5* expression), which in turn inhibits NLRP3 inflammasome-associated pyroptosis, ultimately promoting osteogenesis and improving bone microstructure [61]. Regarding diabetic retinopathy, the negative correlation between irisin expression and disease severity has been elaborated in the preceding text. Irisin may exert a retinal protective effect to counteract the occurrence and progression of DR through anti-inflammatory, anti-angiogenic, and other pathways [24,31].

In summary, as a key molecular bridge linking exercise and health benefits, irisin plays a complex role in the pathological processes of diabetes mellitus and its complications. Existing evidence supports its multi-organ protective properties, including the amelioration of diabetic cardiomyopathy, diabetic nephropathy, diabetic osteopathy, diabetic neuropathy, and diabetic retinopathy.

### 4.6. Irisin in the Retina: Expression, Receptor Distribution, and Multi-Cellular Targeting Potential

The therapeutic promise of irisin in ameliorating DR pathology is closely associated with its intraretinal distribution and the functional expression of receptors on relevant target cells (Table 4). Irisin has been detected in the neural retina and the RPE [31,180], indicating that its local production could complement endocrine signaling from exercise-stimulated peripheral tissues such as skeletal muscle. Additionally, *FNDC5* mRNA, which encodes the irisin precursor, is reported in aqueous humor, serum, and retinal tissue [31,181,182], supporting its probable expression within discrete retinal cell types including photoreceptors, RGCs, and Müller glial cells. This broad expression profile further substantiates the hypothesis of local irisin synthesis and establishes a foundation for exploring irisin-mediated intercompartmental signaling within the retina.

The biological functions of irisin are primarily mediated by integrin receptors, with integrin αVβ5 being the most characterized in the retina. Expression of integrin αVβ5 has been confirmed in vascular endothelial cells [54], RPE cells [55], retinal microglia [56], and astrocytes [57], providing direct evidence for irisin signaling in these cell types and suggesting its potential to modulate diverse retinal cell populations. Based on this receptor distribution, we propose that irisin exerts distinct yet overlapping regulatory effects across different retinal compartments, and that its synergistic actions may mitigate multiple pathological features of DR (Table 2). These compartment-specific effects are likely influenced by localized irisin availability and receptor expression patterns, as well as the unique functional role of each cell type, though further validation of their precise contributions in DR is warranted.

Irisin demonstrates multi-targeted neuroprotective potential in the retina, with mechanisms and contributions that are cell type-specific. At the neuronal level, irisin has been shown in central neuronal models like the hippocampus to demonstrate neuroprotective properties, promote neurogenesis, and regulate synaptic plasticity [27], supporting the likelihood of similar functions in retinal neurons. Within the vascular compartment, irisin contributes to vascular homeostasis by inhibiting endothelial inflammation and reducing oxidative stress [183], effects that help maintain vascular integrity, protect blood-brain barrier function [186], and restrain pathological neovascularization [31]. Based on these systemic actions, we hypothesize that irisin likely operates through analogous pathways in the retinal vasculature. In the glial network, irisin exerts key immunomodulatory actions, promoting a shift in microglia from the M1 to the M2 phenotype [150] and possibly modulating Müller cell activation, which collectively attenuates neuroinflammation and the release of factors such as VEGF. Together, through coordinated regulation of neuronal survival, vascular stability, and glial function, irisin establishes an integrated, multi-layered protective network in the retina, highlighting its promise as a therapeutic candidate and warranting further investigation.

### 4.7. Relative Contribution and Synergy of Local and Distal Irisin in DR Protection

The protective effects of irisin against DR depend on the synergistic interplay between its distally and locally produced forms. Among these, endogenously derived irisin from distal sites acts as an endocrine factor and serves as the cornerstone of systemic retinal protection, with skeletal muscle being its primary source. Exercise activates PGC-1α, which in turn upregulates *FNDC5* expression and promotes irisin cleavage and subsequent release into the circulation [20]. A significant inverse correlation between serum irisin levels and DR severity [23,174] validates the clinical significance of systemic irisin. Circulating irisin can cross the BRB; it exerts anti-inflammatory and antioxidant effects to maintain BRB integrity, ameliorate the systemic pro-diabetic microenvironment, and establish a baseline protective threshold for the retina. However, reduced irisin synthesis in skeletal muscle under diabetic conditions [23,174,187] lowers this protective threshold, thereby increasing the risk of retinal damage.

Locally produced irisin acts in a paracrine/autocrine manner and functions as a precise modulator of retinal protection. Both irisin and its precursor *FNDC5* are endogenously expressed in retinal neurons, vascular endothelial cells, and RPE cells [31,181,182], with their synthesis likely regulated by retinal cellular stress and metabolic status. By operating via paracrine/autocrine signaling pathways, this local irisin elicits rapid responses to local perturbations such as hyperglycemia and oxidative stress. It not only maintains retinal microenvironmental homeostasis and regulates the functions of adjacent cells, but also initiates prompt protective responses through αVβ5 integrin receptors expressed on retinal cells [54,55,56,57], thus compensating for the relatively slow, sustained signaling of circulating irisin.

These two sources of irisin complement each other and collectively mediate the protective effects against DR. Specifically, myokine-derived irisin acts as a core mediator linking exercise to retinal protection, laying a systemic foundation for defending against metabolic disorders and inflammatory injuries. In contrast, retina-derived irisin undertakes the precise regulation of the local microenvironment and maintains retinal homeostasis through regional defense and repair mechanisms.

## 5. Summary and Outlook

This article offers a systematic overview of the neuroprotective effects of irisin in DR and the underlying molecular mechanisms. Accumulating evidence indicates that irisin ameliorates glucose metabolism disorders by activating the AMPK/PI3K/Akt signaling pathway. Moreover, irisin may act by suppressing NF-κB activation and modulating microglia/macrophage polarization, thereby alleviating neuroinflammation and reducing vascular leakage in DR. Notably, irisin demonstrates significant anti-ferroptotic potential, likely mediated via the AMPK/Nrf2/GPX4 axis or the SIRT1p53 pathway. These mechanisms enhance cellular antioxidant defenses and restore iron metabolism homeostasis, thereby inhibiting high-glucose-induced lipid peroxidation and ferroptotic death of retinal cells, ultimately preserving BRB integrity. Clinically, both circulating and intraocular irisin levels are markedly reduced in patients with DR and exhibit a negative correlation with disease severity. This suggests that irisin may function as an endogenous protective factor involved in the pathophysiological progression of DR. However, it is important to note that clinical diagnosis of DR and the efficacy of treatment are exclusively based on the vasculature. Nevertheless, research on irisin in the context of DR remains in its early stages. A key challenge in this field lies in elucidating the differential effects of various exercise modalities on irisin secretion and defining the optimal parameters for its clinical application.

Current clinical therapies for diabetic retinopathy, including anti-VEGF agents and laser photocoagulation, primarily target late-stage vascular lesions, offer insufficient intervention against early neurodegeneration and inflammation, and are often associated with adverse effects. Consequently, their therapeutic efficacy falls short of meeting comprehensive clinical demands. Current evidence regarding irisin’s function in DR is derived primarily from animal studies and in vitro experiments. Its precise protective effects and underlying mechanisms in human diabetic retinopathy have not yet been fully elucidated. To achieve the dual goals of neuroprotection and vascular stabilization, it is essential to further investigate the multi-target effects of irisin. Such research will not only help clarify how exercise provides ocular protection but also offer critical evidence for developing future exercise-based therapeutic approaches. Therefore, it is recommended that individuals with T2DM engage in at least 150 min of moderate-intensity aerobic exercise weekly to aid in preventing or delaying the onset of DR.

To further harness the therapeutic potential of irisin in DR, future research should prioritize the following directions: First, it is essential to systematically analyze the interaction between exercise and individual baseline health status, including factors such as age, glycemic control level, the extent of basic fundus lesions, and the presence of metabolic syndrome. This analysis should clarify the differences in the responses of individuals at high risk of DR and early-stage patients with varying baseline characteristics to irisin exercise, particularly regarding potential “exercise thresholds” or differences in “response sensitivity.” Second, it is crucial to accurately define the optimal exercise parameters that promote retinal neuroprotection. This includes quantitatively grading exercise intensity, such as relative intensity based on maximum oxygen uptake (VO_2_max), as well as determining the duration of exercise, encompassing both single exercise sessions and the total intervention period, and identifying the differential effects of various exercise types, including endurance training, HIIT, and resistance training. It is precisely by addressing these research gaps that we can gain deeper insight into the mechanisms by which irisin regulates retinal homeostasis and establish an evidence-based clinical foundation for developing personalized exercise intervention protocols for DR. This will contribute to the creation of integrated management strategies that combine exercise with pharmacological therapy, thereby more effectively preventing and mitigating the onset and progression of DR.

## Figures and Tables

**Table 1:** Endurance Exercise Effects on Irisin.

Exercise Type	Study Subjects	Gender; Age	Exercise Protocols	Study Outcomes	References
Endurance exercise	Human	males; aged 30 to 49 years	High-intensity aerobic exercise (running, swimming, cycling)	Significantly increased irisin secretion levels	[58]
	Mice, rats	unspecified; six-month-old mice and twelve-month-old rats	4 weeks of swimming training	Significantly increased plasma irisin levels	[59]
	Human	Unspecified	Regular endurance training	Blood irisin concentration twice that of non-exercise	[20]
	Healthy untrained middle-aged men	males; aged 35 to 59 years	High-intensity endurance training	No significant changes in skeletal muscle PGC-1a, *FNDC5*, or serum irisin	[63]
	Healthy middle-aged and elderly	men and women; 67 ± 8 years	8 weeks of endurance training	Serum irisin levels significantly increased	[64]
	Healthy young adults	men and women; 21 ± 1 years	8 weeks of endurance training	No significant effect on serum irisin levels	[64]

**Table 2:** Acute Exercise Effects on Irisin.

Exercise Type	Study Subjects	Gender; Age	Exercise Protocols	Study Outcomes	References
Acute exercise	Human	males; 23 ± 2 years	High-intensity interval training (HIIT), resistance training	No significant difference in serum irisin levels between exercise and control groups	[65]
	Human	men and women; 27.4 ± 3.8 years	Exhaustive running (high-intensity exercise)	Induced irisin release increases both during exercise and recovery	[68]
	Human	men and women; 27.4 ± 3.8 years	Exhaustive cycling (high-intensity exercise)	Weak promoting effect on irisin release	[68]
	Obese adolescents	males; 15.4 ± 1.1 years	Skipping rope (HIIT form), combined with dark chocolate supplementation	Significantly increased irisin secretion, improved body composition and inflammatory markers	[69]
	Human	males; 18.4 ± 0.1 years	Soccer match (morning, afternoon, evening)	Serum irisin levels significantly increased	[70,71]
	Human	males; 21.64 ± 1.22 years, women; 22.64 ± 1.49 years	20-s all-out cycling sprint, observed at 15/30/60 min recovery	Irisin concentration significantly increased at all recovery time points	[72]
	Human	men; 20.5 ± 1.5 years	Consecutive HIIT	Increased *FNDC5* mRNA expression in skeletal muscle	[73]
	Human	female; aged 14 to 18 years	Consecutive HIIT	No significant difference in plasma irisin expression	[74]
	Human	males; 24 + 2 years	Endurance exercise alone, resistance exercise alone, combined resistance + endurance exercise	Resistance exercise-induced irisin response better than endurance alone, combined intervention optimal	[75]

**Table 3:** Resistance Exercise Effects on Irisin.

Exercise Type	Study Subjects	Gender, Age	Exercise Protocols	Study Outcomes	References
Resistance exercise	Human	males; 29.37 ± 5.14 years	8 weeks of resistance training	No significant effect on serum irisin levels	[76]
	Human	males; 62 years	12 weeks of lower limb and core high-intensity intermittent static training (e.g., leg raises) resistance training	Increased serum irisin levels	[77]
	C57BL/6 mice	males; fourteen months	12 weeks of resistance training	Increased serum irisin levels	[78]
	Human	men and women; unspecified	Single session high-intensity strength training (e.g., weightlifting), observed 1 h post-training	Blood irisin concentration transiently increased, peaked at 1 h post-training, then returned to baseline	[79]
	Human	males; unspecified	Single session weight training	No significant change in irisin levels	[80]
	Human	women; 21.00 ± 1.33 years	Compound resistance exercises like squats, push-ups	Induced irisin secretion	[81]

**Table 4:** Irisin/*FNDC5* and Integrin Receptor Expression and Function in Retinal Cells.

Retinal Cell Type	Expression of Irisin/*FNDC5*	Expression of Irisin Receptors (Integrins)	Primary Function	References
Ganglion Cells	Expression	Undefined	Anti-apoptosis, anti-oxidative stress.	[31,56]
Vascular Endothelial Cells	Expression	αVβ5	Anti-inflammatory, anti-oxidative stress, anti-apoptosis, inhibit pathological neovascularization.	[31,54,183]
Astrocytes	Undefined	αVβ5	Protection against apoptosis, anti-inflammatory, inhibit pathological neovascularization. (inferred from studies on non-retinal astrocytes)	[31,57,184,185]
Microglia	Undefined	αVβ5	Polarization from M1 to M2 phenotype, reduced neuroinflammation.	[56,150]
RPE Cells	Expression	αVβ5	Antioxidative stress, maintenance of phagocytic and trophic functions.	[31,55]

## Data Availability

No new data were created or analyzed in this study. Data sharing is not applicable to this article.
